# Adult presentation of Bartter syndrome type IV with erythrocytosis

**DOI:** 10.1590/S1679-45082015RC3013

**Published:** 2015

**Authors:** Ita Pfeferman Heilberg, Cláudia Tótoli, Joaquim Tomaz Calado

**Affiliations:** 1 Universidade Federal de São Paulo, São Paulo, SP, Brazil.; 2 Faculdade de Ciências Médicas, Universidade Nova de Lisboa, Lisboa, Portugal.

**Keywords:** Bartter syndrome, Hypokalemia, Chloride channels, Case reports

## Abstract

Bartter syndrome comprises a group of rare autosomal-recessive salt-losing disorders with distinct phenotypes, but one unifying pathophysiology consisting of severe reductions of sodium reabsorption caused by mutations in five genes expressed in the thick ascending limb of Henle, coupled with increased urinary excretion of potassium and hydrogen, which leads to hypokalemic alkalosis. Bartter syndrome type IV, caused by loss-of-function mutations in barttin, a subunit of chloride channel CLC-Kb expressed in the kidney and inner ear, usually occurs in the antenatal-neonatal period. We report an unusual case of late onset presentation of Bartter syndrome IV and mild phenotype in a 20 years-old man who had hypokalemia, deafness, secondary hyperparathyroidism and erythrocytosis.

## INTRODUCTION

Antenatal Bartter syndrome (BS) type IV is characterized by polyhydramnios, premature birth, sensorineural deafness, severe salt and water loss, in the perinatal period, hypokalemic alkalosis, fever, vomiting, diarrhea, failure to thrive and chronic renal failure developing during infancy.^(1)^ Late-onset presentation of BS with sensorineural deafness caused by a mutation of barttin has already been described in Japan,^(2)^ Portugal^(3)^ and Spain^(4)^ but to the best of our knowledge, this is the first case described in Brazil.

## CASE REPORT

A deaf male patient was referred to the Nephrology unit for the first time at the age of 20 years due to asthenia and severe hypokalemia (potassium − K of 2.0mEq/L; normal range: 3.5 to 5.0mEq/L), accompanied by erythrocytosis (hemoglobin − Hb of 18.7g/dL; normal range: 12 to 15.5g/dL) detected at preoperative tests preceding a knee operation. The patient was the tenth child of consanguineous parents, who born premature by cesarean-section delivery after a complicated pregnancy by polyhydramnios. The patient had other deaf brother and a stone-forming sister. His parents noticed the severe hypoacusia on his second year of life. Upon admission, the patient was normotensive (110/70mmHg), had serum creatinine of 0.9mg/dL (normal range: 0.6 to 1.2mg/dL), weight of 54kg and 1.50m height (below the fifth percentile stature for age). Intravenous potassium chloride (KCI) replacement was started with 19.1% 20mL, followed by oral KCI supplementation 6.0% 20mL t.i.d. Additional serum laboratorial determinations showed a serum bicarbonate of 23.0mmoL/L, slightly decreased serum ionized calcium (1.09mmoL/L; normal range: 1.15 to 1.32mmoL/L), low serum phosphate (2.2mg/dL; normal range: 2.5 to 4.5mg/dL) and reduced fractional tubular reabsorption of phosphate (TRP; 74.1%; normal: >80%) and high serum intact parathyroid hormone (PTH; 120ng/L; normal range: 15 to 68ng/L). Serum 25OH - vitamin D (28.2ng/mL) was slightly below the normal ranges (>30ng/mL). Plasma renin (65.0ng/mL; upper limit <6.0ng/mL) and aldosterone (55.7ng/dL; upper limit <31ng/dL) were increased. His urinary volume was 3,530mL/day but hypercalciuria was not detected. A computed helical tomography excluded nephrocalcinosis. Urinary retinol-binding protein (RBP; 41mg/L; upper limit <0.40mg/L) was markedly elevated. The etiology of erythrocytosis was investigated. Leucocytes and platelet counts were normal (and the bone marrow biopsy was mildly hypocellular except for an erythroid hyperplasia). Serum iron, ferritin and transferrin were normal. Erythropoietin (EPO) was also within normal limits (19.2mUI/mL). Oxyhemoglobin dissociation curve (P50) was normal and Janus kinase 2 (JAK-2) mutation analysis was negative, ruling out polycythemia vera. Two weeks after hospital discharge, spironolactone (100mg/day) was added to the oral KCI supplementation (30mEq/day). During follow-up the spironolactone dose was increased to 200mg/day for a better control of hypokalemia.

## DISCUSSION

Although the finding of mild hypophosphatemia and elevated serum intact PTH could have initially suggested the presence of some disorder of phosphate metabolism in the present case, the association of hypokalemia and hearing impairment, even in the absence of metabolic alkalosis, led us to hypothesize about a late onset presentation of BS type IV. The diagnosis was confirmed by molecular analysis disclosing a c.139G>A allele for the BSND gene in homozygosity, resulting in glycine (Gly) to arginine (Arg) at position 47 (p.G47R).

As pointed out by Brum et al.,^(3)^ it is possible that the co-expression of p.G47R barttin and CLC-Ka may result in a less severe reduction of chloride currents, as seen in missense mutations, enabling barttin to retain some residual function with CLC-Kb, conditioning a milder phenotype.^(5)^ Indeed, in a recent functional study, Janssen et al.^(6)^ have shown that the G47R was the only missense mutation tested that did not prevent the insertion of barttin into the surface membrane nor the activation of CLC-Kb/barttin channels, but that it impairs expression levels and complex glycosylation of the CLC-Kb channel so that its binding by barttin turns to be less effective. Therefore, distinct mutations of BSND cause phenotypes of varying severity.

In the present case, renal function was preserved, like in all other described patients carrying this mutation. The absence of metabolic alkalosis in the current patient although unexpected, has already been described in cases of BS type I or II^(7,8) ^or even in other adult onset presentations of BS type IV.^(2)^

The most intriguing feature of the present case was the presence of a marked erythrocitosis in a non-smoking patient, in the absence of polycythemia vera, JAK-2 mutations or other causes of primary polycythemia. One case of Bartter associated with erythrocytosis had already been described in the literature in 1973 by Erkelens,^(9)^ who hypothesized that the observed elevated erythropoietc activity of the serum could have resulted from juxtaglomerular hyperplasia leading to overproduction of both renin and EPO. However, the major source of EPO synthesis in the kidney is presently known to be the interstitial fibroblasts and not the juxtaglomerular apparatus. Besides, EPO levels showed to be within normal range in the present case. Although the erytrocitosis might have been secondary to polyuria, the 24 hours urine volume of the current patient was not so high to cause volume contraction. Therefore, the exact cause of erytrocytosis remains unclear.

Increased levels of serum PTH could have been ascribed to mild hypocalcemia but not to hypomagnesemia, which was not observed in the present case. Pseudohypoparathyroidism (PHP) has been reported in patients with BS.^(10,11)^ However, the observed low levels of serum phosphate, due to a reduced TRP do not suggest PHP. These findings are in agreement with Vaisbich et al.,^(12)^ who also reported hypophosphatemia in 5 out of 12 BS cases. Finally, after a 2-month course of oral cholecalciferol supplementation (50,000UI), PTH levels normalized, suggesting that high PTH might have been secondary to the mild hypocalcemia and sub-normal levels of 25OH - vitamin D.

In addition to phosphaturia, another evidence of proximal tubular dysfunction in the current case was the increased level of urinary RBP, a low molecular weight protein. Although the etiology of such dysfunction cannot be fully understood, other case has already been reported in the literature consisting of an adult onset Fanconi syndrome with kidney medullary cystic disease, non-specific aminoaciduria, lysozymuria and beta2-microglobulinuria, hyperreninemia and polycythemia with elevated serum EPO levels resembling Bartter’s syndrome.^(13)^ Another characteristic phenotype usually described in BS cases is the hypercalciuria, associated or not to nephrocalcinosis. Our patient did not show this phenotype, which agrees with other reports^(2,3)^ possibly because of the low serum ionized calcium that led to a lower filtered load of calcium.

## CONCLUSION

Molecular diagnosis is significant for a better understanding of the pathophysiology and approach to treatment of renal tubular disorders such as Bartter due to the phenotypic heterogeneity seen in this syndrome.
