# Supplementary material for: Adult presentation of Bartter syndrome type IV with erythrocytosis
Source: Einstein (Sao Paulo). 2015 Oct-Dec;13(4):604–6. doi: 10.1590/S1679-45082015RC3013 (PMC4878638; doi:10.1590/S1679-45082015RC3013)
Supplement: Translation

# Translation: Apresentação tardia de síndrome de Bartter tipo IV com eritrocitose

**Authors:** Ita Pfeferman Heilberg, Cláudia Tótoli, Joaquim Tomaz Calado

**Affiliations:** 1 Universidade Federal de São Paulo, São Paulo, SP, Brasil.; 2 Faculdade de Ciências Médicas, Universidade Nova de Lisboa, Lisboa, Portugal.

**Keywords:** Síndrome de Bartter, Hipopotassemia, Canais de cloreto, Relatos de casos

## Abstract

A síndrome de Bartter compreende um grupo raro de doenças autossômicas recessivas perdedoras de sal, decorrentes de mutações em genes expressos na porção ascendente espessa da alça de Henle, com fenótipos distintos, porém fisiopatogenia única, que consiste em redução severa da reabsorção de sódio, e aumento da excreção urinária de hidrogênio e potássio, levando à alcalose hipocalêmica. A síndrome de Bartter tipo IV, causada por mutações com perda de função da bartina, uma subunidade do canal de cloro CLC-Kb expressa no rim e ouvido interno, geralmente se apresenta nos períodos ante e neonatal. No presente relato, descreve-se um caso não usual de síndrome de Bartter tipo IV com apresentação tardia e fenótipo atenuado, diagnosticado por análise molecular, em um homem adulto de 20 anos que se apresentava com hipocalemia, surdez, hiperparatireoidismo secundário e eritrocitose.

## INTRODUÇÃO

A síndrome de Bartter (SB) antenatal tipo IV é caracterizada por polihidrâmnio, nascimento prematuro, *déficit* auditivo sensório-neural e perda severa de sal e água no período perinatal, alcalose hipocalêmica, febre, vômito, diarreia, *déficit* de crescimento e desenvolvimento de insuficiência renal crônica na infância.^(1) ^A apresentação de SB de início tardio com perda auditiva sensório-neural causada por mutação no gene da bartina já foi descrita no Japão,^(2) ^em Portugal^(3)^ e na Espanha,^(4)^ mas trata-se do primeiro caso descrito no Brasil.

## RELATO DE CASO

Paciente surdo, do sexo masculino, foi encaminhado para a enfermaria de Nefrologia pela primeira vez aos 20 anos de idade devido a quadro de astenia e hipocalemia aguda (potássio − K sérico de 2,0mEq/L; valor normal de 3,5 a 5,0mEq/L), acompanhados de eritrocitose (hemoglobina − Hb de 18,7g/dL; valor normal de 12 a 15,5g/dL) detectados em exame pré-operatórios anteriores a uma cirurgia de joelho. Tratava-se do décimo filho de pais consanguíneos, que nasceu prematuro de parto cesárea, após gestação complicada por polihidrâmnio. O paciente apresentava um outro irmão com surdez e uma irmã com cálculo renal. Seus pais notaram sua hipoacusia no segundo ano de vida. Na internação, o paciente apresentava-se normotenso (110/70mmHg), com creatinina sérica de 0,9mg/dL (valor normal de 0,6 a 1,2mg/dL), peso de 54kg e altura de 1,50m (abaixo do percentil 5 de estatura para idade). Iniciou-se reposição intravenosa com cloreto potássio (KCI) 19,1% 20mL intravenoso, seguido por suplementação de KCI via oral 6,0% 20mL três vezes ao dia. Além disso, as determinações laboratoriais séricas mostraram bicarbonato sérico de 23,0mmoL/L, redução discreta de cálcio sérico ionizado (1,09mmoL/L; valor normal de 1,15 a 1,32mmoL/L), fosfato sérico baixo (2,2mg/dL; valor normal de 2,5 a 4,5mg/dL), redução da reabsorção tubular fracional de fósforo (TRP - *tubularreabsorption of phosphate*; 74,1%; normal acima de 80%) e paratormônio (PTH) intacto elevado (120ng/L; valor normal de 15 a 68ng/L). Os níveis séricos de 25OH-vitamina D (28,2ng/mL) estavam discretamente abaixo do normal (>30ng/mL). Foram observadas elevações na renina plasmática (65,0ng/mL; limite normal <6,0ng/mL) e aldosterona (55,7ng/dL; limite normal <31ng/dL). O volume urinário do paciente era de 3.530mL/dia, porém não foi detectada hipercalciuria. Excluiu-se nefrocalcinose, por meio de tomografia computadorizada helicoidal. A proteína ligadora de retinol (RBP - *retinol-binding protein*) (41mg/L; limite normal <0,40mg/L) mostrou-se significativamente elevada. A etiologia dos eritrocitose também foi investigada. A contagem de plaquetas e leucócitos encontrava-se normal (biópsia da medula óssea revelou-se discretamente hipocelular com exceção da hiperplasia eritroide). Ferro sérico, ferritina e transferrina, bem como eritropoietina (EPO), encontravam-se dentro dos limites de normalidade (19,2mUl/mL). A curva de dissociação da oxihemoglobina (P50) estava normal, e a análise para mutação da Janus-quinase 2 (JAK-2) foi negativa, sendo descartada policitemia vera. Na alta hospitalar após 2 semanas, foi prescrita espironolactona (100mg/dia), além da suplementação com KCI por via oral (30mEq/dia). No seguimento, a dose de espironolactona foi aumentada para 200mg/dia, para melhor controle da hipocalemia.

## DISCUSSÃO

No presente caso, apesar de os achados de hipofosfatemia leve com PTH intacto elevado poderem ter sugerido inicialmente a presença de algum distúrbio do metabolismo de fósforo, a associação de hipocalemia e deficiência auditiva, mesmo na ausência de alcalose metabólica, levou à hipótese de SB tipo IV com início tardio. O diagnóstico foi confirmado por meio de análise molecular, que revelou um alelo c.139G>A para o gene BSND em homozigose, resultando na troca de glicina (Gly) para arginina (Arg) na posição 47 (p.G47R).

Conforme descrito por Brum et al.,^(3)^ é possível que a coexpressão do p.G47R de bartina, com os canais CLC-Ka, tenha resultado em uma redução menos severa das correntes de cloro, como observado em mutações *missense*, possibilitando que a bartina retivesse algumas funções residuais, como CLC-Kb, condicionando um fenótipo menos severo.^(5)^ No entanto, em um estudo funcional recente, Janssen et al.^(6) ^demonstraram que a G47R foi a única mutação *missense* examinada que não preveniu a inserção da bartina na superfície da membrana, nem a ativação dos canais CLC-Kb/bartina, porém tal mutação alterou os níveis de glicosilação dos canais CLC-Kb, de forma que sua ligação por meio de bartina se tornasse menos efetiva. Portanto, mutações distintas na bartina causam fenótipos com diferentes níveis de severidade.

Neste caso, a função renal do paciente estava preservada, corroborando todos os casos descritos na literatura de pacientes com a mesma mutação. A ausência de alcalose metabólica em nosso paciente, apesar de inesperada, já foi descrita em casos de SB tipos I e II,^(7,8) ^e até nos primeiros sintomas da SB tipo IV em adultos.^(2)^

A característica mais intrigante de nosso caso foi a presença de uma marcada eritrocitose (poliglobulia) em um paciente não fumante, na ausência de policitemia vera, mutações na JAK-2 ou de outras causas de policitemia primária. Um caso de Bartter associado com eritrocitose já foi descrito na literatura em 1973 por Erkelens,^(9)^ que supôs que a elevada atividade eritropoiética no soro poderia ter resultado de uma hiperplasia juxtaglomerular*,* levando à produção excessiva tanto de renina quanto de EPO. Porém considera-se atualmente que a maior fonte de síntese de EPO no rim sejam os fibroblastos intersticiais, e não o aparelho juxtaglomerular. Além disso, no presente caso, os níveis séricos de EPO encontravam-se dentro dos limites da normalidade. Apesar de a eritrocitose poder ter sido secundária à poliúria, o volume de urina de 24 horas nesse paciente não estava elevado a ponto de causar uma contração de volume. Assim, a causa exata da eritrocitose permanece desconhecida.

Os níveis elevados de PTH poderiam ser atribuídos à hipocalcemia leve, mas não à hipomagnesemia, não observada no presente caso. Associação com pseudo-hipoparatireoidismo (PHP) tem sido relatada em pacientes com SB.^(10,11)^ Porém, os níveis baixos de fosfato sérico observados, devido à reduzida reabsorção tubular, não sugerem PHP. Esses achados corroboram os de Vaisbich et al.,^(12)^ que também relataram hipofosfatemia em 5 de 12 casos com SB. Por fim, após 2 meses de suplementação com colecalciferol por via oral (50.000UI), os níveis de PTH se normalizaram, sugerindo que o PTH elevado deve ter sido secundário à hipocalcemia leve e aos níveis subnormais de 25OH-vitamina D.

Além da fosfatúria, outra evidência de disfunção tubular proximal no presente caso foi o aumento do nível urinário de proteína de baixo peso molecular, como a proteína ligadora de retinol (RBP). Apesar da etiologia dessa disfunção não estar completamente esclarecida, um outro caso de síndrome de Fanconi de início na idade adulta, associado a doença renal cística medular, aminoacidúria inespecífica, lisozimúria, perda de beta2-microglobulina, hiperreninemia e policitemia, com níveis elevados de eritropoetina sérica similares aos da SB, já foi descrito na literatura.^(13)^ Outra característica fenotípica geralmente descrita nos casos de SB é a hipercalciúria, associada ou não a nefrocalcinose, que não foi detectada em nosso paciente, também corroborando outros relatos,^(2,3)^ possivelmente devido ao cálcio sérico reduzido, que leva a menor carga filtrada.

## CONCLUSÃO

O diagnóstico molecular foi importante para entender melhor a fisiopatogenia de distúrbios tubulares renais, tal como a síndrome de Bartter, em função da heterogeneidade fenotípica presente nessa síndrome.
